# Fusion Pore: A Curious Case of Rediscovering Science

**DOI:** 10.15190/d.2017.3

**Published:** 2017-04-16

**Authors:** Nodar P. Mitagvaria

**Affiliations:** I. Beritashvili Center of Experimental Biomedicine, 14 Gotua Street, 0160 Tbilisi, Georgia

**Keywords:** Fusion Pore, SNARE ring complex, membrane fusion, Porosome - the secretory machinery

In pioneering studies, the group of James E. Rothman discovered that target membrane proteins called t-SNAREs and vesicle-associated membrane proteins called v-SNAREs are the minimal fusion machinery involved in cells. In a recent review^1^entitled: “*Fusion pores* and their control of neurotransmitter and hormone release”, the authors present in Figure 2 a model of the t-/v-SNARE rosette or ring complex, completely ignoring earlier studies hypothesizing this model^2^(see Figure 5) and the actual experimental confirmation of the model^3-5^ (Figure 2C^3^; Figure 2^4^ and Figure 2^5^) nearly two decades ago. This is how science reinvents itself and pioneering contributions are knowingly ignored, hoping that the rest of the scientific community will remain ignorant of earlier findings and discoveries. In 1998, the group of James E. Rothman published in the journal Cell including on the cover, an elegant model of the sagittal section of a t-SNARE and v-SNARE mediated secretory vesicle docked at the target membrane, demonstrating the rosette or ring confirmation of the assembly of the t-/v-SNARE complex^2^ (Figure 5). This elegant assembly of the SNARE complex in a rosette published by James E. Rothman has been experimentally demonstrated using both atomic force microscopy^3-5^ and electron microscopy^6^, by Bhanu P. Jena and his research team. The SNARE complexed as a rosette or ring has also been hypothesized and modeled by many groups, including the late Ilan Hammel^7^. Curiously, in the recent *J. Gen. Physiol.* 2017 Vol. 149 No. 3 301–322 review article^1^, there is no discussion, mention, or reference of any kind to these earlier published findings.

 Similarly, in the past 20 years following discovery of the supramolecular secretory machinery in cells, the “*porosome”*^8,9^, there has been a flood of papers on new protein being identified to associate with t-SNAREs at the so called “fusion protein complex” or the “fusion active zone” at the cell plasma membrane^10^, completely ignoring the actual *porosome* structure at the cell plasma membrane where secretory vesicles transiently dock and fuse to release a fraction of their contents in a highly regulated manner, as opposed to an all-or-none mechanism of complete vesicle merger at the cell plasma membrane. Seeing is believing, and the physical existence of the porosome using atomic force microscopy, and confirmed by electron microscopy and X-Ray solution imaging is undeniable. Scores of studies continue to be reported on the role of various porosome proteins on cell secretion and their altered states resulting in secretory defects – *a tale of rediscovering science*.

## References

[1] Chang Che-Wei, Chiang Chung-Wei, Jackson Meyer B (2017). Fusion pores and their control of neurotransmitter and hormone release.. The Journal of general physiology.

[2] Weber T, Zemelman B V, McNew J A, Westermann B, Gmachl M, Parlati F, Söllner T H, Rothman J E (1998). SNAREpins: minimal machinery for membrane fusion.. Cell.

[3] Cho Sang-Joon, Kelly Marie, Rognlien Katherine T., Cho Jin Ah, Heinrich Hörber J.K., Jena Bhanu P. (2002). SNAREs in Opposing Bilayers Interact in a Circular Array to Form Conducting Pores. Biophysical Journal.

[4] Cho Won Jin, Jeremic Aleksandar, Jena Bhanu P (2005). Size of supramolecular SNARE complex: membrane-directed self-assembly.. Journal of the American Chemical Society.

[5] Jeremic Aleksandar, Quinn Anthony S, Cho Won Jin, Taatjes Douglas J, Jena Bhanu P (2006). Energy-dependent disassembly of self-assembled SNARE complex: observation at nanometer resolution using atomic force microscopy.. Journal of the American Chemical Society.

[6] Cho Won Jin, Lee Jin-Sook, Zhang Lei, Ren Gang, Shin Leah, Manke Charles W, Potoff Jeffrey, Kotaria Nato, Zhvania Mzia G, Jena Bhanu P (2011). Membrane-directed molecular assembly of the neuronal SNARE complex.. Journal of cellular and molecular medicine.

[7] Hammel Ilan, Meilijson Isaac (2013). Function suggests nano-structure: towards a unified theory for secretion rate, a statistical mechanics approach.. Journal of the Royal Society, Interface.

[8] Lee Jin-Sook, Jeremic Aleksandar, Shin Leah, Cho Won Jin, Chen Xuequn, Jena Bhanu P (2012). Neuronal porosome proteome: Molecular dynamics and architecture.. Journal of proteomics.

[9] Naik Akshata R, Lewis Kenneth T, Jena Bhanu P (2016). The neuronal porosome complex in health and disease.. Experimental biology and medicine (Maywood, N.J.).

[10] Wickner William, Rizo Josep (2017). A cascade of multiple proteins and lipids catalyzes membrane fusion.. Molecular biology of the cell.

